# Pilot Randomized Controlled Trial of *Lymfit*: A Theory-Guided Exercise Intervention for Young Adults with Lymphoma

**DOI:** 10.3390/healthcare12111101

**Published:** 2024-05-28

**Authors:** Wing Lam Tock, Nathalie A. Johnson, Ross E. Andersen, Matthew Salaciak, Christopher Angelillo, Carmen G. Loiselle, Maude Hébert, Christine Maheu

**Affiliations:** 1 Ingram School of Nursing, Faculty of Medicine and Health Sciences, McGill University, Montréal, QC H3A 2M7, Canada; carmen.g.loiselle@mcgill.ca (C.G.L.); christine.maheu@mcgill.ca (C.M.); 2 Division of Experimental Medicine, Faculty of Medicine and Health Sciences, McGill University, Montréal, QC H4A 3J1, Canada; nathalie.johnson@mcgill.ca; 3 Department of Medicine, Jewish General Hospital, Montréal, QC H3T 1E2, Canada; 4 Department of Kinesiology and Physical Education, Faculty of Medicine and Health Sciences, McGill University, Montréal, QC H2W 1S4, Canada; ross.andersen@mcgill.ca (R.E.A.); christopher.angelillo@mail.mcgill.ca (C.A.); 5 Department of Medicine, Faculty of Medicine and Health Sciences, McGill University, Montréal, QC H3G 2M1, Canada; matthew.salaciak@mail.mcgill.ca; 6 Department of Oncology, Faculty of Medicine and Health Sciences, McGill University, Montréal, QC H4A 3T2, Canada; 7 Département des Sciences Infirmières, Université du Québec à Trois-Rivières, Trois-Rivières, QC G8Z 4M3, Canada; maude.hebert@uqtr.ca

**Keywords:** young adult cancer survivors, lymphoma, exercise motivation, exercise intervention, self-determination theory, pilot feasibility study, randomized controlled trial

## Abstract

Despite the rapidly emerging evidence on the contributions of physical activity to improving cancer-related health outcomes, adherence to physical activity among young adults with lymphoma remains suboptimal. Guided by self-determination theory (SDT), the *Lymfit* intervention (a 12-week individualized exercise program with bi-weekly kinesiologist support and an activity tracker) aimed to foster autonomous motivation toward physical activity. This pilot randomized controlled trial aimed to evaluate the feasibility, acceptability, and preliminary effects of *Lymfit*. Young adults (N = 26; mean age of 32.1 years) with lymphoma who were newly diagnosed and those up to six months after completing treatment were recruited and randomly assigned one-to-one to either the intervention group (n = 13) or a wait-list control group (n = 13). All a priori feasibility benchmarks were met, confirming the feasibility of the study in terms of recruitment uptake, retention, questionnaire completion, intervention fidelity, missing data, Fitbit wear adherence, and control group design. The intervention acceptability assessment showed high ratings, with eight out of ten items receiving >80% high ratings. At post-intervention, an analysis of covariance models showed a clinically significant increase in self-reported physical activity levels, psychological need satisfaction, and exercise motivation in the intervention group compared to controls. *Lymfit* also led to meaningful changes in six quality-of-life domains in the intervention group, including anxiety, depression, fatigue, sleep disturbance, social roles and activities, and pain interference. The findings support *Lymfit* as a promising means to meet psychological needs and increase the autonomous motivation for physical activity in this group. A fully powered efficacy trial is warranted to assess the validity of these findings.

## 1. Background

Young adults (YAs) aged 18–39 are considered one of the fastest-growing segments of cancer survivors in Canada [1]. Lymphoma, cancer of the lymphatic system, is a commonly diagnosed cancer affecting YAs [2,3]. Lymphoma can be highly curable with chemotherapy and/or radiotherapy; however, these treatments can have potentially serious short- and long-term adverse effects [4]. For instance, anthracycline-based regimens and mediastinal/thoracic radiation therapy can increase the risk of cardiovascular and pulmonary complications and radiation-induced hypothyroidism [5,6]. Besides the treatment-induced long-term effects, YAs with lymphoma encounter a variety of psychological and functional challenges upon the completion of their cancer treatments. These challenges include cancer-related fatigue [7] and decreased cognitive capability [8], both of which can lead to decreased productivity and quality of life [9,10]. Furthermore, according to a longitudinal, population-based survey conducted in the Netherlands, YAs with lymphoma reported more psychological distress (e.g., anxiety and depression) and a lower quality of life compared to the general population [8,11]. Consequently, cancer diagnoses and their treatment can significantly hamper the productive years of YAs [9]. 

Yet, the long-term supportive needs among YA with lymphoma remain understudied [12]. Physical activity is a promising means to reduce the intensity and frequency of toxicities resulting from cancer treatment agents, along with enhancing both physical and psychosocial health among cancer survivors [13,14]. Evidence suggests that post-diagnosis physical activity reduces all-cause and cancer-specific mortality among survivors of breast, prostate, and colorectal cancers [15]. Physical activity among individuals with lymphoma is also shown to significantly modulate psychological distress and illness-related anxiety [16], improve quality of life [17], alleviate fatigue [18], and prolong survival [19,20], in addition to promoting cardiovascular health and muscle strength [21].

Despite a substantial body of evidence demonstrating the positive impact of physical activities on cancer-related health outcomes, adherence to recommended physical activity guidelines among cancer survivors remains sub-optimal [22]. Notably, lack of motivation is a frequently reported psychological barrier to physical activity engagement in this population [23]. The literature suggests that focusing on modifying the motivational factors in health behavior interventions can yield multiple positive effects for cancer survivors, such as improved quality of life, the restoration of order in life, and the preservation of meaning to life in the face of illness and health challenges [24]. 

Self-determination theory (SDT) is a macro-theory of human motivation [25,26]. SDT is relevant to understanding the mechanisms of health behavior change, including the maintenance of exercise [27,28,29]. SDT provides a framework for intervention development by proposing that three basic psychological needs (i.e., autonomy, competence, and relatedness) must be supported to foster autonomous forms of motivation (or intrinsic motivation) [30], which, in turn, are associated with important health outcomes, including psychological health, well-being, and improved quality of life [31]. Accordingly, exercise interventions based on or informed by SDT have grown considerably in recent years [32]. However, limitations to these SDT-informed interventions exist, for example, (a) the physical activity program is predetermined or standardized and is not tailored to cancer survivors’ needs (lack of autonomy support), (b) key components of competence support (e.g., goal-setting) are neglected, and/or (c) the measurement and interpretation of results are not conducted in relation to the theory [33,34]. To address these limitations, we have developed and pilot-tested *Lymfit*—an individualized, virtually delivered, and SDT-guided intervention aiming to promote exercise motivation in YAs who have been diagnosed with and treated for lymphoma.

**Figure 1:**
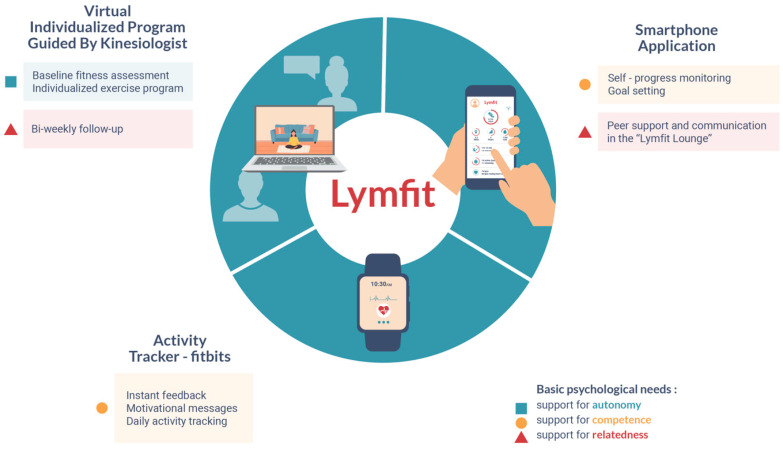
*Lymfit* intervention components.

*Lymfit* is a 12-week, virtually delivered, and individualized exercise intervention. Theoretically guided by SDT, *Lymfit* is designed to enhance the motivation for exercise engagement in YA with lymphoma by providing support in the three basic psychological needs (Figure 1). The intervention participants are given a Fitbit, which provides functions such as task orientation, goal-setting, progress monitoring, and feedback (support for competence). Participants are prescribed a personalized 12-week exercise program by the kinesiologist, which is tailored to their baseline fitness level and exercise tolerance (support for autonomy). The progress of the intervention participants is followed by the study kinesiologist for 12 weeks (with bi-weekly follow-up consultations), and they are also connected with other intervention participants within the “*Lymfit lounge*”, a private group on the Fitbit smartphone application where participants can share and compare their exercise progress and activity achievements (support for relatedness).

The development of *Lymfit* has been an iterative process. The preliminary version of *Lymfit* was reviewed with YA lymphoma survivors for initial feedback. Then, we recently undertook a proof-of-concept study early on in the development of the intervention, in which 20 long-term YA lymphoma survivors participated in a single-armed pilot study that aimed to examine the implementation feasibility (e.g., technical and safety issues) of the preliminary version of *Lymfit* [35].

Overall, the novelty of the *Lymfit* intervention lies in its innovative combination of individualization, flexibility, and theory-informed motivational techniques, offering a promising approach to enhancing exercise engagement among YAs with lymphoma. This present study aimed to pilot-test *Lymfit* through a randomized controlled trial (RCT). Specifically, the objectives of this pilot RCT were to assess *Lymfit*’s (a) feasibility through predetermined a priori benchmarks; (b) its acceptability; and (c) its preliminary effects on four study outcomes: psychological need satisfaction, exercise motivation, physical activity level, and health-related quality of life.

## 2. Methods

### 2.1. Design

This study was a 1:1, parallel, two-group (intervention and wait-list control group) pilot RCT (clinical trial registration: NCT05259657). The design and reporting of this study were guided by the Consolidated Standards of Reporting Trials (CONSORT) 2010 guideline for randomized pilot and feasibility trials [36] (Table S1 in the Supplementary Materials) and the Template for Intervention Description and Replication (TIDieR) guideline [37] (Table S2 in the Supplementary Materials). This study was approved by the Research Ethics Boards from the two recruiting sites in Montreal, Quebec. 

### 2.2. Setting, Recruitment, Participants, and Sample Size

Study participants were recruited from two university-affiliated hospitals in Montréal, Canada, recommended by hematologists and by self-referral via flyers in the oncology clinic from February to November 2022. Newly diagnosed YAs with lymphoma aged 18 to 39 who had a score of <14 (classified as sedentary) on the Godin–Shephard leisure-time physical activity questionnaire (LTPA-Q) [38] were considered eligible. (A cutoff score of ≥24 on the LTPA-Q classifies the participants as active, a score between 14 to 23 is classified as moderately active, and a score of <14 is classified as sedentary [38]. The LTPA-Q is a commonly used tool for classifying cancer survivors into active and sedentary categories [39]. Participants also had to: be either receiving or having completed chemotherapy within the past six months; own a smartphone; and have an internet connection at home. The power calculation for sample size was not performed for this study. Instead, based on the recommendations for pilot RCTs, a target sample size of at least 12 per group was set [40,41]. 

### 2.3. Randomization and Blinding

**Figure 2:**
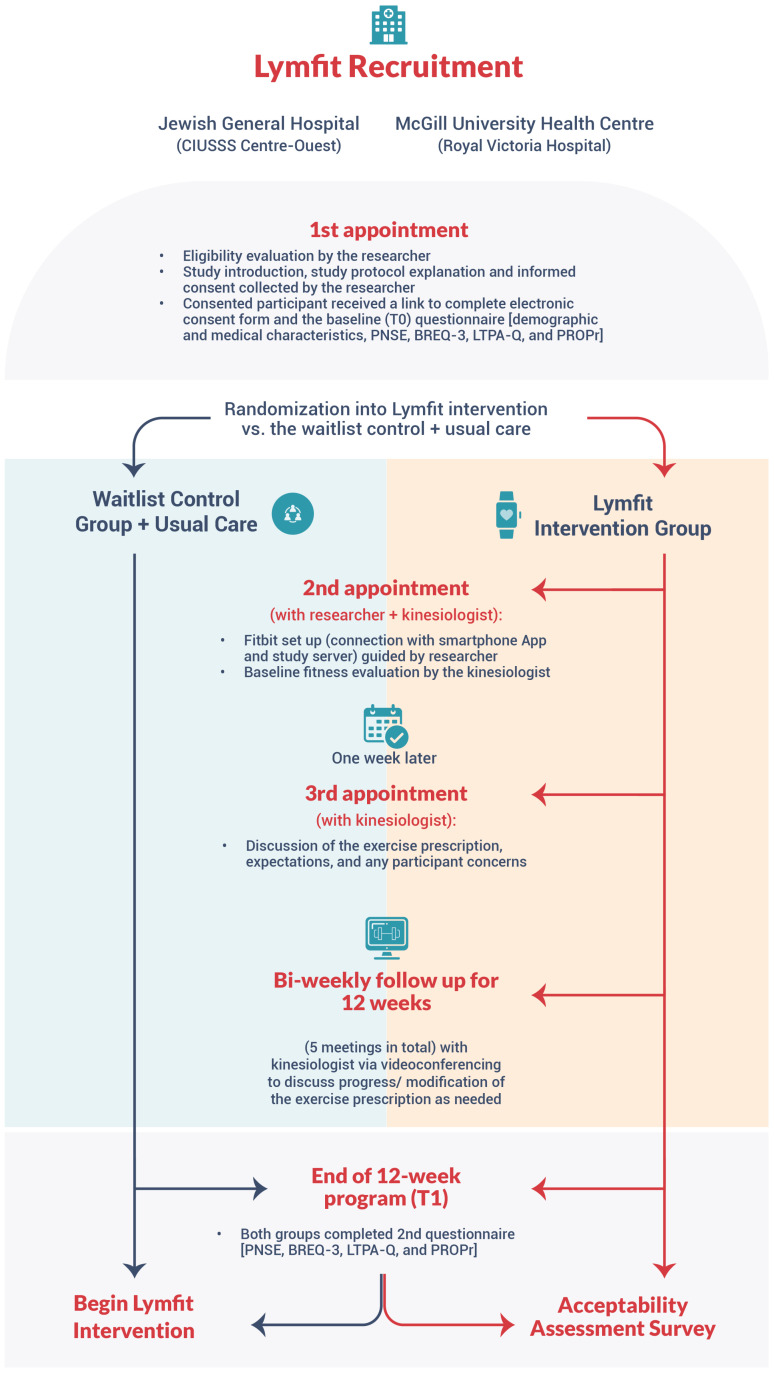
*Lymfit* study procedures. Note. PNSE = psychological need satisfaction in exercise; BREQ-3 = behavioral regulation in exercise questionnaire; LTPA-Q = Godin–Shephard leisure-time physical activity questionnaire; PROPr = patient-reported outcome measurement information system^®^—preference.

The study coordinator scheduled the first study appointment with eligible participants via videoconferencing (study procedures are shown in Figure 2). During this meeting, the participants completed an electronic consent form and baseline measures (i.e., T0), comprising questions on demographics and medical characteristics and a set self-reported questionnaire. The study coordinator then registered the participants’ pre-assigned Fitbit (Charge V model, Fitbit Inc., San Francisco, CA, USA) on the *Lymfit* platform (study web database), which randomized participants to the intervention or the control group using a computer-generated randomization schedule stratified according to chemotherapy completion status (i.e., completed chemotherapy vs. undergoing chemotherapy). To ensure allocation concealment and avoid selection bias, the *Lymfit* platform was programmed by a statistician who was not involved in the study, and research team members did not have access to the randomization schedule.

### 2.4. Study Groups

Intervention group. All study appointments were conducted via videoconferencing. First, the study package consisted of a pre-assigned Fitbit, and then exercise resistance bands were mailed to the participants. At the second study appointment, the study coordinator guided each participant in setting up their Fitbit and pairing it with their smartphone application. All participants were then added to a virtual “*Lymfit lounge*”, acting as a peer-support group within the Fitbit application. During the same appointment, the study kinesiologist conducted a baseline physical assessment for each participant (details previously published in the proof-of-concept study of *Lymfit* [35]). In the following week, the kinesiologist evaluated the data collected from the assessment. Using the baseline data, the kinesiologist established an individualized exercise program for the participant. One week after the second appointment, the study kinesiologist met with the participant to discuss the exercise program, expectations, and any participant concerns (third study appointment). 

Each individualized exercise program is designed around the evidence-based exercise guidelines targeting cancer survivors published by the American College of Sports Medicine [14], while taking into consideration the results from the baseline assessment for each participant. In summary, the exercise program was guided by the FITT principles: a minimum of 3 times per week (Frequency); at a moderate-to-vigorous level (Intensity); for 30 min each session, for 12 weeks (Time); and with aerobic activity favored over resistance training (Type) [14]. The exercise program in this study incorporated individualized and incremental aspects of goal-setting. These operate upon two main assumptions: firstly, by individualizing the goals, the goals are more specific to an individual’s lifestyle and are, thus, more achievable for them, enhancing their autonomy. Secondly, incrementally introducing the exercise program over the course of 12 weeks would make it less difficult for participants to adjust to their goals as it would be less cognitively demanding and impactful on participants’ lifestyles, especially considering that these are individuals who are undergoing or have just completed cancer treatments. 

Thereafter, follow-up appointments with the study kinesiologist were conducted every 2 weeks for a duration of 12 weeks. During these sessions, participants engaged in discussions with the kinesiologist to review their progress and make the necessary modifications or advancements to their exercise programs. At the end of the 12-week intervention (i.e., T1), participants completed the outcome measures and an acceptability assessment survey. Participants were instructed to complete these measures within one week of intervention completion. 

Wait-list control group. Control-group participants continued their usual practices as per the recruiting sites’ protocol. Once the outcome measures were completed at T1, the study kinesiologist contacted the participants in the wait-list control group to begin the *Lymfit* intervention. The study coordinator documented whether control-group participants remained in the study at the second kinesiologist follow-up meeting (i.e., T2).

### 2.5. Data Collection

Demographic and clinical characteristics. Questions on demographic and clinical characteristics were part of the baseline assessment questionnaire completed by all participants before randomization at T0. 

Feasibility. A set of a priori benchmarks was established to determine the feasibility of the *Lymfit* intervention. The a priori benchmarks for recruitment and retention rate were at least 50% [42,43] and 70% [35], respectively. The a priori benchmark for questionnaire completion is to have at least 95% of study participants complete the questionnaires at both T0 and T1 [35]. For intervention fidelity, we aimed to have 90% of sessions delivered in accordance with the fidelity checklist [42]. Regarding missing data, less than 10% of missing data on the study questionnaires was considered as meeting the benchmark [44]. Fitbit wear adherence is defined as the percentage of days in the 12-week intervention period that the participants logged a valid day of wear. A Fitbit wear day is considered valid if more than 1000 step counts are logged during that day [45]. The a priori benchmark for adherence is at least 85% of valid days over the 12-week intervention [46]. Lastly, the control group design is considered feasible if 90% of the wait-list control-group participants started and remained in the intervention at T2. 

A study log was kept by the study coordinator to collect data on feasibility criteria throughout the study. For instance, data concerning recruitment and retention rates (e.g., the number of patients approached, the number of self-referred patients, the number of eligible and ineligible patients, the number of patients who declined to participate (with reasons), the number of participants consenting, and randomized) were documented in the study log. 

Acceptability. An acceptability assessment survey, comprising 10 items tailored to *Lymfit*, was collected at T1 from the intervention group. Participants rated their satisfaction with each intervention component and the suitability of delivery procedures on a 5-point Likert scale, with a higher score indicating a more positive endorsement of the statement. 

Self-reported outcomes. The preliminary effects of the *Lymfit* intervention on the four self-reported study outcomes were assessed through questionnaires collected from all participants at baseline, both before randomization (T0) and post-intervention (T1). A detailed description and the psychometric properties of the instruments can be found in Table S3 in the Supplementary Materials. 

The psychological need satisfaction in exercise (PNSE) scale [47] was used to assess the perception of psychological need satisfaction associated with exercise motivation (18 items in total). The overall satisfaction scores and the three subscale scores (perceived support for competence, autonomy, and relatedness) can be calculated, with higher scores indicating greater need satisfaction.

Exercise motivation (i.e., self-determination) was assessed using the behavioral regulation in exercise questionnaire (BREQ-3) [48,49]. The BREQ-3 comprises 24 items and 6 subscales, measuring the 6 types of motivations (i.e., amotivation, external regulation, introjection, identification, integration, and intrinsic regulation). The subscale scores were weighted to provide an overall estimate of self-determination, the relative autonomy index (RAI), for which higher scores reflect more self-determination (more exercise motivation).

The self-reported physical activity level was assessed using the three-item Godin–Shephard leisure-time physical activity questionnaire (LTPA-Q) [38]. The LTPA-Q asks individuals to recall the number of times in the past 7 days that they have performed any strenuous, moderate, or mild/light physical activity of more than 15 min in duration. A total physical activity score can be calculated.

The patient-reported outcomes measurement information system^®^—Preference (PROPr) [50] was used to measure the perceived quality of life in eight domains (i.e., physical function, anxiety, depressive symptoms, fatigue, sleep disturbance, ability to participate in social roles and activities, pain interference, and cognitive function, comprising 30 items in total). A T-score was generated from each subscale, with higher scores indicating greater endorsement of the construct being assessed [51]. A PROPr utility score (representing overall quality of life) was also calculated [50]. 

### 2.6. Data Analysis

Demographics, feasibility, and acceptability data. Participant characteristics, feasibility, and acceptability data were summarized using descriptive statistics. Baseline group equivalence was assessed using appropriate statistical tests. For feasibility data, percentages pertaining to the criteria (e.g., recruitment uptake, retention rate, questionnaire completion, etc.) were calculated and compared to the a priori benchmarks. For the acceptability survey items, a score of 4 or 5 on a 5-point Likert scale (i.e., 4 = acceptable, 5 = highly acceptable) is considered a high rating. The percentage of high ratings for each question was reported.

Preliminary effects. All data analyses were conducted on R Studio (v. 2023.09.1+494). Independent *t*-tests were used to compare the mean values of all self-reported study outcomes at baseline between the intervention and control groups. Analysis of covariance (ANCOVA) models were used to compare post-intervention group differences for the study outcomes between the two groups, where the post-intervention values of the study outcomes were the dependent variables, the baseline (pre-intervention) values served as covariates, and the grouping variable identified the two study groups. Assumption checks for all data on the self-reported outcomes were first checked using appropriate statistical tests and plots. The normality of the variables was tested using the Shapiro-Wilk test. Two outcome variables (BREQ-3, Integrated regulation) and (PROPr, physical function) violated the normality assumption (*p* = 0.014; *p* = 0.040 respectively). Logarithmic transformation was applied to these two variables and the assumption was met afterward (*p* = 0.228, *p* = 0.059, respectively). 

Univariate models were used to examine the homogeneity of regression assumption on each dependent variable. Full-factor ANCOVA models were then fitted to evaluate the group differences in the post-intervention scores adjusted for the baseline scores. For this pilot investigation, an effect size of at least 0.2 for each study outcome was considered acceptable [52]. Additionally, the minimal important change (MIC) was calculated for quality-of-life domains (measured by PROPr), striving for a threshold of 4 T-score points change to be considered a meaningful within-group change and between-group comparison [53,54]. 

## 3. Results

### 3.1. Participant Characteristics

**Figure 3:**
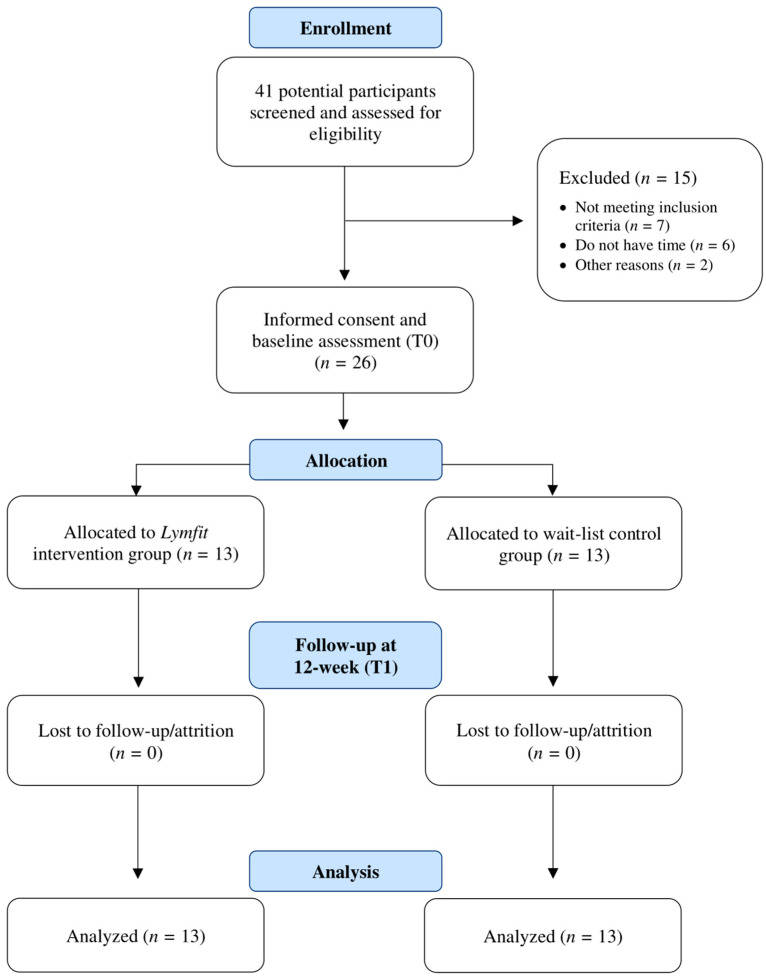
Consolidated standards of reporting trials (CONSORT) flow diagram.

A total of 26 YAs with lymphomas were randomized. The mean age of the participants was 32.4 years old (SD = 5.82, range = 20–39). The majority of the study participants were female (84.6%) and white (92.3%), and more than half had some university or college education (57.7%). Approximately half of the participants were married (53.8%) and did not have dependent children (57.7%). One-third were employed or going to school full-time. The mean body mass index (BMI) was 24.93 kg/m^2^ (SD = 4.35, range = 16.86–34.87), which is considered within the healthy weight range [55]. Nearly one-third were undergoing chemotherapy. Equal numbers of participants were diagnosed with Hodgkin’s lymphoma and non-Hodgkin’s lymphoma and were receiving chemotherapy in the frontline setting with curative intent (Table 1). The Figure 3 CONSORT flow diagram details the participants’ flow through the study.

### 3.2. Feasibility

**Figure 4:**
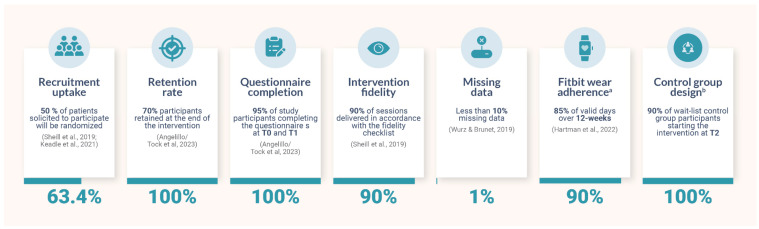
A priori feasibility benchmarks and results [35,42,43,44,46]. Note: ^a^ Data collected in the intervention group only (n = 13). ^b^ Data collected in the control group only (n = 13).

All predetermined feasibility benchmarks were achieved (Figure 4). Of the 41 potential participants screened for eligibility, 26 were enrolled and randomized into the 2 study groups, representing a 63.4% recruitment uptake rate. The retention and questionnaire completion rates were both 100%, with minimal (<1%) data missing. For Fitbit wear adherence (n = 13), valid wear days over the 12-week intervention period were 90% (982 of 1092 total days). Furthermore, no protocol infringements occurred during the study, and 90% of the sessions were delivered in accordance with the fidelity checklist. Reasons for missed follow-up appointments included: participants going on vacation, sickness, being unable to schedule a meeting time due to school or work obligations, etc. For most of the missed follow-up appointments, the study kinesiologist was able to connect with the participants via email or phone calls to discuss program progress and to address any concerns from the participants. Finally, all participants randomized to the wait-list control group were successfully retained in the study at T2.

### 3.3. Acceptability

The thirteen participants from the intervention group completed the acceptability assessment survey at T1. Item number five, which assessed participant enjoyment of using the peer-support group on the smartphone application, received the least number of high ratings (53.8%). This represents a low acceptability of the peer support group component. Additionally, 23% rated item number three (Was the frequency of the kinesiologist follow-up acceptable?) with a score of three or below. The rest of the items received >80% high ratings (i.e., 4 or 5 on a 5-point scale). Item #10 assessed the participant’s overall satisfaction with the *Lymfit* intervention and received 92.3% high ratings (Table 2). 

### 3.4. Preliminary Effects on Study Outcomes 

Table 3 presents the ANCOVA results of the four self-reported outcome measures. The T0 values, T1 adjusted values, the effect size (ES) with 95% CI, and *p*-values are presented. In addition, post hoc power analysis was conducted for each of the main study outcomes using G*Power (Version 3.1.9.7) (Table S3 in the Supplementary Materials). It should be noted that our preliminary results for power analysis should be interpreted with caution. The benchmark for effect size, which was at least 0.2, was mostly met for the self-reported study outcomes. As hypothesized, the intervention group participants reported improvements in all four main study outcomes (ES of overall PNSE = 0.498, ES of overall BREQ-3 = 0.598, ES of LTPA-Q = 0.348, and ES of overall PROPr = 0.332).

The ANCOVA model showed a group effect on overall psychological need satisfaction at post-intervention after adjusting for baseline score differences (overall PNSE: *p* < 0.001, intervention: *M* = 3.96, *SE* = 0.06, vs. control: *M* = 3.52, *SE* = 0.06). Among the three PNSE subscales, the competency and autonomy subscales met the ES threshold of 0.2 (ES = 0.255; 0.311), while the relatedness subscale did not meet the ES threshold (ES = 0.128). 

For self-determination (exercise motivation, as measured by BREQ-3), the ANCOVA model showed a significant group effect on overall self-determination at post-intervention after adjusting for baseline score differences (overall BREQ-3: *p* < 0.001, intervention: *M* = 4.50, *SE* = 0.63, vs. control: *M* = −0.85, *SE* = 0.63). Among the six subscales, amotivation was the only one meeting the ES threshold of 0.2 (ES = 0.636). Next, the result of the ANCOVA showed that there was a significant group effect on self-reported physical activity levels at post-intervention (LTPA-Q: *p* = 0.002, intervention: *M* = 40.36, *SE* = 3.67, vs. control: *M* = 22.06, *SE* = 3.67) after controlling for the participants’ score at baseline. Lastly, the ANCOVA model showed a significant group effect on overall quality of life at post-intervention after adjusting for baseline score differences (PROPr: *p* = 0.003, intervention: *M* = 0.53, *SE* = 0.05, vs. control: *M* = 0.29, *SE* = 0.05). The ES threshold was also met for four out of the eight PROPr subscales, including physical function (ES = 0.385), fatigue (ES = 0.346), ability to participate in social roles and activities (ES = 0.291), and pain interference (ES = 0.355). 

In terms of PROPr domains (Table 4), six subscales out of eight exhibited beneficial changes in T-scores that met the MIC threshold of a minimum of four T-score points over time in the intervention group (i.e., anxiety, depression, fatigue, sleep disturbance, ability to participate in social roles and activities, and pain interference). Furthermore, seven subscales out of eight met the MIC threshold for between-group comparisons (i.e., physical function, anxiety, depression, fatigue, and sleep disturbance, the ability to participate in social roles and activities, and pain interference). 

## 4. Discussion

Overall, this pilot RCT garnered promising findings. The *Lymfit* intervention addresses the need to promote exercise motivation among YAs undergoing lymphoma treatment or immediately post-treatment. The study documented key benchmarks for feasibility, acceptability, and the preliminary effects of the intervention, in preparation for a larger trial.

Several key findings are worth noting. First, this pilot study tested *Lymfit* using a rigorous design and demonstrated the feasibility and acceptability of the intervention. All a priori feasibility benchmarks were met. The results provide a strong foundation for future testing on a larger scale. In particular, the wait-list control design is highly feasible; a 100% retention rate in the control group was achieved. These findings are consistent with the literature, suggesting that a wait-list control group can improve retention as compared to usual care/no-treatment control groups in exercise interventions [56]. 

Another main significance of this study is that it demonstrated the acceptability of virtual exercise intervention delivery during treatment and immediately post-treatment. This finding suggests that rehabilitation could be implemented in conjunction with cancer therapy to enhance the quality of life of YAs affected by lymphoma [57].

Furthermore, promising trends were found for all the main outcome variables, including overall psychological need satisfaction, overall exercise motivation, physical activity levels, and overall quality of life. Among all the self-reported outcomes, the largest effect size of 0.636 was observed for the amotivation subscale (measured using the BREQ-3), the least desirable type of motivation as posited by the SDT. These preliminary results suggest that *Lymfit* has a significant effect on moving the intervention group participants up the relative autonomy continuum from amotivation. In addition, the MIC threshold was met for multiple domains in the PROPr for both within-group changes and between-group differences post-intervention, demonstrating the positive effects of the intervention on the participants’ quality of life. The above findings are in line with SDT, reflecting the significance of providing a favorable environment for performing exercise during and immediately after cancer treatment, supporting the autonomy, competence, and relatedness required for health behavior change [29].

With regard to psychological need satisfaction (measured with the PNSE), our preliminary findings show that the intervention had significant effects on competence and autonomy, but not on relatedness. This is concordant with another study’s findings, which show a low acceptability of the use of peer-support groups. The low utilization of the peer-support group may be driven by the fact that participation in the in-app *Lymfit lounge* was not mandatory. Further modification to the support of relatedness needs is required. For instance, social support from family and friends was probably another essential aspect of the cancer rehabilitation interventions highlighted in the literature that may be added to future interventions [58]. 

Our intervention has several strengths. First, despite the absence of face-to-face participant interaction, our study results demonstrated the promising effects of our virtually delivered intervention. A virtual exercise intervention offers several advantages, including increased accessibility and convenience for participants, particularly for individuals undergoing active cancer treatment who may face logistical challenges in attending in-person exercise training sessions. In addition, *Lymfit* provided flexible, individualized programs tailored to YA cancer survivors’ unique needs. Compared to standardized or group-formatted interventions, individually tailored interventions can better provide autonomy support [59]. In accordance with the literature, physical activity interventions should be tailored for personal facilitators, barriers, and motivations to maximize survivorship adaptations [60]. A recent systematic review of physical activity interventions in pediatric, adolescent, and YA cancer survivor populations reported that the majority of the studies were focused on pediatric and adolescent populations, missing the opportunity to examine the effects of exercise in YAs [61]. Furthermore, none of the interventions in this review offered individualized programs or comprehensive content to address psychological needs. Of note, the recruitment rate and retention rate in the present pilot study exceeded those intervention studies that are included in this review, which might endorse the more flexible intervention design adopted by *Lymfit*.

This study has some limitations. First, more women than men agreed to participate in the study. Although this is commonly reported in exercise intervention studies [62], more inclusive recruitment strategies are warranted for a more diverse sample in future trials. Another potential bias related to eligible patients who declined to participate in the study because of time constraints is acknowledged, and it indicates that recruitment strategies need to be developed in future trials to address potential participants’ concerns. For instance, we should reinforce the notion that this exercise program is tailored to each participant’s availability and needs; therefore, participation in the study should not conflict with their daily routine. The potential for co-intervention bias poses an additional limitation. This bias could arise from additional interventions received by participants outside the study intervention (e.g., attending gym class or hiring a private trainer), which might potentially confound the outcome of interest [63]. Future trials should monitor the use of additional resources and any exercise engagements (e.g., asking participants to self-report the recourses used at each data-collection time point). Such monitoring would facilitate the identification of co-intervention effects, enable appropriate adjustments during the analysis, and provide for a more unbiased interpretation of the study results.

Future studies should consider employing a fully powered trial with a longitudinal design to assess the effectiveness of *Lymfit* over time. Such investigations can incorporate additional exploratory analyses to identify mediating and moderating factors for physical activities. Regression analyses can further elucidate specific factors, such as psychological needs or behavior regulation styles, that may predict the maintenance of physical activity levels and quality-of-life outcomes [64].

Future studies could rely on a mixed-method approach, which may offer valuable insights into the barriers and facilitators associated with participation in exercise interventions among YAs with lymphoma. This approach can also address the gaps identified in our pilot RCT, including the identification of strategies to enhance social connectedness and to determine the optimal frequency of kinesiologist consultations for patients undergoing chemotherapy and those who have completed treatment. Such information can be invaluable for clinicians and researchers to tailor exercise programs that better meet the unique needs of this patient population.

## 5. Conclusions

This pilot RCT was considered successful, given that feasibility, acceptability, and the promising preliminary effects of the intervention were supported. The generally positive outcomes can be attributable to several factors. First, the development of *Lymfit* has been an iterative process, with continuous input from YAs with lymphoma. Second, *Lymfit* guides YAs through the behavior change process, supported by a powerful theoretical framework, which sets it apart from many other exercise interventions. Third, an individualized exercise program delivered during cancer treatment and immediately after cancer treatment might be practical for patients with low motivation and limited experience in exercising. *Lymfit* has the potential to address the fundamental importance of motivational support in exercise interventions by focusing on satisfying basic psychological needs. If further corroborated, SDT-guided interventions may be more broadly implemented to promote exercise engagement and quality of life among cancer survivors.

In summary, the evidence from this pilot RCT can guide the selection of main outcomes and secondary outcomes for larger trials and identify those areas in need of improvement for a larger trial, as mentioned above, such as the format of relatedness support. Even if the results of this pilot study are promising, a larger trial needs to be conducted prior to concluding that *Lymfit* is effective. 

## Figures and Tables

**Table 1:** Participant demographic and clinical characteristics.

	Intervention (n = 13)	Control (n = 13)	Overall (N = 26)
	Mean (Range) ± SD/ n (%)	Mean (Range) ± SD/ n (%)	Mean (Range) ± SD/ n (%)
Age (years)	30.69 (24–39) ± 5.78	34.0 (20–39) ± 5.58	32.35 (20–39) ± 5.82
BMI	25.11 (18.29–32.85) ± 4.56	24.76 (16.86–34.87) ± 4.32	24.93 (16.86–34.87) ± 4.35
BMI categories			
<18.5	1 (7.7)	1 (7.7)	2 (7.7)
18.5–24.9	6 (46.2)	7 (53.8)	13 (50.0)
25.0–29.9	3 (23.1)	3 (23.1)	6 (23.1)
≥30.0	3 (23.1)	2 (15.4)	5 (19.2)
Gender			
Female	11 (84.6)	11 (84.6)	22 (84.6)
Male	2 (15.4)	2 (15.4)	4 (15.4)
Ethnicity/Racial identity			
White	12 (92.3)	12 (92.3)	24 (92.3)
Black	0 (0.0)	1 (7.7)	1 (3.8)
Asian	1 (7.7)	0 (0.0)	1 (3.8)
Education			
High school or less	0 (0.0)	1 (7.7)	1 (3.8)
High school graduate	0 (0.0)	0 (0.0)	0 (0.0)
Some CEGEP	0 (0.0)	0 (0.0)	0 (0.0)
Some university/college	7 (53.8)	8 (61.5)	15 (57.7)
College/technician school degree	0 (0.0)	0 (0.0)	0 (0.0)
University degree	5 (38.5)	4 (30.8)	9 (34.6)
Graduate degree	1 (7.7)	0 (0.0)	1 (3.8)
Marital status			
Married/Common law	7 (53.8)	7 (53.8)	14 (53.8)
Divorced/Separated	0 (0.0)	1 (7.7)	1 (3.8)
Single	6 (46.2)	4 (30.8)	10 (38.5)
In partnership	0 (0.0	1 (7.7	1 (3.8
Household income (CAD)			
$30,000–$60,000	3 (23.1)	4 (30.8)	7 (26.9)
$60,001–$90,000	7 (53.8)	8 (61.5)	15 (57.7)
$90,001–$120,000	3 (23.1)	1 (7.7)	4 (15.4)
Employment/Education			
Full-time	6 (46.2)	4 (30.8)	10 (38.5)
Part-time	2 (15.4)	3 (23.1)	5 (19.2)
Full-time homemaker	0 (0.0)	3 (23.1)	3 (11.5)
On leave	5 (38.5)	3 (23.1)	8 (30.8)
Number of dependent children			
0	8 (61.5)	7 (53.8	15 (57.7
1	1 (7.7)	3 (23.1)	4 (15.4)
2	2 (15.4)	2 (15.4)	4 (15.4)
3 or more	2 (15.4)	1 (7.7)	3 (11.5)
Chemo status			
Not yet completed	4 (30.8)	5 (38.5)	9 (34.6)
Completed	9 (69.2)	8 (61.5)	17 (65.4)
Diagnosis			
Hodgkin’s lymphoma	5 (38.5)	8 (61.5)	13 (50.0)
Non-Hodgkin’s lymphoma	8 (61.5)	5 (38.5)	13 (50.0)

Note. There were no significant differences between the two groups at baseline.

**Table 2:** Acceptability assessment survey results.

Questionnaire Items	High Ratings ^a^
	n (%)
How helpful was the personalized exercise program from the kinesiologist in motivating you to exercise?	11 (84.6)
2. Are you satisfied with the remote format of the exercise program?	11 (84.6)
3. Was the frequency of the kinesiologist follow-up meetings acceptable?	10 (76.9)
4. How helpful was wearing the Fitbit tracker and receiving alerts in motivating you to exercise?	12 (92.3)
5. How much did you enjoy using the peer-support group on the app?	7 (53.8)
6. How helpful was the progress monitoring function on the app in motivating you to exercise?	11 (84.6)
7. Was the amount of time it took to complete this program (12 weeks) acceptable?	13 (100.0)
8. Was the exercise program prescribed by the kinesiologist tailored to your personal needs?	12 (92.3)
9. Was starting this exercise program so close to completing your cancer treatment acceptable?	11 (84.6)
10. How would you rate your overall satisfaction with the *Lymfit* program?	12 (92.3)

Note. ^a^ Items were assessed with a 5-point Likert scale ranging from 1 to 5, with a higher score indicating a more positive endorsement of the statement. A score of 4 or 5 is considered a high rating.

**Table 3:** Analysis of the covariance results.

Instruments and Outcomes	Baseline		Post-Intervention (Adjusted)		Effect Size	*p*-Value ^a^
	Intervention (n = 13)	Control (n = 13)	Intervention (n = 13)	Control (n = 13)		
	Mean ± SD	Mean ± SD	Mean ± SD	Mean ± SD	ηp^2^	
			(95% CI)	(95% CI)	(95% CI)	
Psychological Need Satisfaction in Exercise (PNSE)						
Overall need satisfaction	3.62 ± 0.47	3.44 ± 0.38	3.96 ± 0.22	3.52 ± 0.22	0.498	<0.001**
			(3.82, 4.09)	(3.38, 3.65)	(0.181–0.671)	
Competence	3.20 ± 0.80	3.21 ± 0.73	3.62 ± 0.32	3.24 ± 0.32	0.255	0.01 *
			(3.42, 3.82)	(3.05, 3.42)	(0.016–0.489)	
Autonomy	4.05 ± 0.61	4.00 ± 0.63	4.32 ± 0.40	4.01 ± 0.72	0.311	0.004 *
			(4.08, 4.55)	(3.57, 4.04)	(0.040–0.535)	
Relatedness	3.62 ± 0.65	3.11 ± 0.80	3.94 ± 0.58	3.49 ± 0.58	0.128	0.079
			(3.60, 4.28)	(3.15, 3.84)	(0–0.372)	
Behavioral Regulation in Exercise Questionnaire—Version 3 (BREQ-3)						
Overall self-determination	1.40 ± 5.03	−3.10 ± 5.28	4.50 ± 2.27	−0.85 ± 2.27	0.589	<0.001 **
			(3.20, 5.80)	(−2.15, 0.46)	(0.283–0.732)	
Amotivation	1.50 ± 0.69	1.87 ± 0.93	0.84 ± 0.36	1.77 ± 0.36	0.636	<0.001 **
			(0.63, 1.05)	(1.56, 1.98)	(0.344–0.763)	
External regulation	1.17 ± 0.70	1.10 ± 0.68	0.85 ± 0.54	1.15 ± 0.54	0.077	0.178
			(0.55, 1.16)	(0.84, 1.45)	(0–0.313)	
Introjected regulation	1.54 ± 0.95	2.08 ± 0.87	1.89 ± 0.47	2.21 ± 0.47	0.114	0.098
			(1.62, 2.15)	(1.94, 2.48)	(0–0.357)	
Identified regulation	1.71 ± 0.66	1.58 ± 0.92	2.12 ± 0.47	1.80 ± 0.47	0.115	0.098
			(1.85, 2.39)	(1.53, 2.07)	(0–0.357	
Integrated regulation	1.56 ± 0.69	1.10 ± 0.83	2.03 ± 0.47	1.57 ± 0.47	0.199	0.025 *
			(1.75, 2.30)	(1.30, 1.84)	(0–0.441)	
Intrinsic regulation	1.65 ± 0.76	1.00 ± 0.68	1.53 ± 0.25	1.30 ± 0.25	0.176	0.037 *
			(1.38, 1.67)	(1.16, 1.44)	(0–0.420)	
Godin–Shephard leisure-time physical activity questionnaire (LTPA-Q)						
Self-reported activity level	12.92 ± 5.01	11.04 ± 7.91	40.36 ± 13.23	22.06 ± 13.23	0.348	0.002 *
			(32.77, 47.95)	(14.47, 29.66)	(0.061–0.563)	
Patient-Reported Outcomes Measurement Information System^®^—Preference (PROPr)						
Overall quality of life ^b^	0.28 ± 0.12	0.26 ± 0.10	0.53 ± 0.18	0.29 ± 0.18	0.332	0.003 *
			(0.43, 0.63)	(0.19, 0.39)	(0.051–0.551)	
Physical function ^c^	48.79 ± 7.57	50.15 ± 12.04	52.14 ± 5.84	43.57 ± 5.84	0.385	<0.001 **
			(48.79, 55.50)	(40.22, 46.93)	(0.084–0.590)	
Anxiety	60.59 ± 10.22	60.88 ± 5.19	55.09 ± 7.39	60.21 ± 7.39	0.119	0.091
			(50.72, 59.43)	(55.74, 64.80)	(0–0.362)	
Depression	56.62 ± 10.25	54.57 ± 8.86	50.79 ± 6.16	56.54 ± 6.16	0.196	0.027 *
			(47.26, 54.33)	(53.01, 60.08)	(0–0.439)	
Fatigue	56.84 ± 8.03	58.66 ± 5.93	50.05 ± 7.43	60.25 ± 7.43	0.346	0.002 *
			(45.80, 54.30)	(56.00, 64.50)	(0.060–0.562)	
Sleep disturbance	55.87 ± 4.40	56.77 ± 5.49	46.11 ± 7.43	52.76 ± 7.43	0.184	0.032 *
			(41.85, 50.37)	(48.50, 57.02)	(0–0.428)	
Social roles and activities	48.56 ± 5.75	43.76 ± 5.15	53.68 ± 6.38	45.64 ± 6.38	0.291	0.005 *
			(50.02, 57.34)	(41.99, 49.30)	(0.031–0.519)	
Pain interference	48.79 ± 7.57	50.99 ± 11.77	43.77 ± 6.34	52.64 ± 6.34	0.355	0.002 *
			(40.13, 47.40)	(49.01, 56.27)	(0.065–0.569)	
Cognitive abilities	47.47 ± 7.10	44.52 ± 8.24	49.13 ± 6.13	47.54 ± 6.13	0.018	0.518
			(45.62, 52.64)	(44.03, 51.05)	(0–0.214)	

Notes. ^a^ * *p* = < 0.05; ** *p* = < 0.001 (significance levels between the adjusted means score of the control and experimental groups after intervention). ^b^ Overall quality-of-life score is the PROMIS-Preference (PROPr) score, which provides a preference-based summary score for health states defined by 7 PROMIS domains. It ranges from −0.022 to 1.00. ^c^ PROPr sub-domain values are reported as T-scores with a mean of 50.

**Table 4:** Quality of life domains—minimal important changes (MIC).

PROPr Domains ^a^	Baseline (T0)		Post-Intervention (T1)		Change in T-Score	
	Intervention (n = 13)	Control (n = 13)	Intervention (n = 13)	Control (n = 13)	Intervention (n = 13)	Control (n = 13)
	Mean ± SD	Mean ± SD	Mean ± SD	Mean ± SD	T-Score Change from T0 to T1	
Physical function	48.79 ± 7.57	50.15 ± 12.04	52.18 ± 5.68	43.53 ± 5.82	+3.39	−6.62
Anxiety	60.59 ± 10.22	60.88 ± 5.19	55.02 ± 10.34	60.28 ± 5.69	−5.57	−0.60
Depression	56.62 ± 10.25	54.57 ± 8.86	51.12 ± 6.94	56.22 ± 6.56	−5.50	+1.65
Fatigue	56.84 ± 8.03	58.66 ± 5.93	49.48 ± 8.53	60.82 ± 8.44	−7.36	+2.16
Sleep disturbance	55.87 ± 4.40	56.77 ± 5.49	45.73 ± 8.50	53.14 ± 8.26	−10.14	−3.63
Social roles and activities	48.56 ± 5.75	43.76 ± 5.15	55.48 ± 7.01	43.84 ± 7.41	+6.92	+0.08
Pain interference	48.79 ± 7.57	50.99 ± 11.77	43.48 ± 4.84	52.92 ± 8.12	−5.31	+1.93
Cognitive abilities	47.47 ± 7.10	44.52 ± 8.24	49.98 ± 9.11	46.69 ± 5.15	+2.51	+2.17

Notes ^a^ PROPr sub-scale (domain) values are reported as T-scores with a mean of 50. Green = beneficial change; Red = detrimental change.

## Data Availability

The original data presented in the study are openly available in the OSF data depository at doi: 10.17605/OSF.IO/N5ABK.

## Supplementary Materials

The following supporting information can be downloaded at: https://www.mdpi.com/article/10.3390/healthcare12111101/s1, Table S1: CONSORT 2010 checklist of information to include when reporting a pilot for feasibility trial [36]. Table S2: The template for the intervention description and replication (TIDieR) checklist. Table S3: Description of the study outcome measures and their psychometric properties [38,39,47,48,49,50,65,66,67,68].
