# Supplementary material for: Pilot Randomized Controlled Trial of Lymfit: A Theory-Guided Exercise Intervention for Young Adults with Lymphoma
Source: Healthcare (Basel). 2024 May 28;12(11):1101. doi: 10.3390/healthcare12111101 (PMC11171874; doi:10.3390/healthcare12111101)
Supplement: Supplementary file 1 [file healthcare-12-01101-s001.zip › healthcare-2971496-supplementary.pdf]

Supplementary materials

Table S1 *CONSORT 2010 checklist of information to include when reporting a pilot or feasibility trial*

Section/ Topic	Item No	Checklist item	Reported on page No/ section *
**Title and abstract**			
	1a	Identification as a pilot or feasibility randomised trial in the title	Study title
	1b	Structured summary of pilot trial design, methods, results, and conclusions (for specific guidance see CONSORT abstract extension for pilot trials)	Abstract
**Introduction**			
Background and objectives	2a	Scientific background and explanation of rationale for future definitive trial, and reasons for randomised pilot trial	p 2-3
	2b	Specific objectives or research questions for pilot trial	p 3
**Methods**			
Trial design	3a	Description of pilot trial design (such as parallel, factorial) including allocation ratio	p 3-4
	3b	Important changes to methods after pilot trial commencement (such as eligibility criteria), with reasons	NA
Participants	4a	Eligibility criteria for participants	p 4
	4b	Settings and locations where the data were collected	p 4
	4c	How participants were identified and consented	p 4
Interventions	5	The interventions for each group with sufficient details to allow replication, including how and when they were actually administered	p 5-6 & Figure 2
Outcomes	6a	Completely defined prespecified assessments or measurements to address each pilot trial objective specified in 2b, including how and when they were assessed	P 6-7
	6b	Any changes to pilot trial assessments or measurements after the pilot trial commenced, with reasons	NA
	6c	If applicable, prespecified criteria used to judge whether, or how, to proceed with future definitive trial	p 6-7
Sample size	7a	Rationale for numbers in the pilot trial	p 4
	7b	When applicable, explanation of any interim analyses and stopping guidelines	N/A
Randomisation:			
Sequence generation	8a	Method used to generate the random allocation sequence	p 4
	8b	Type of randomisation(s); details of any restriction (such as blocking and block size)	p 4
Allocation, concealment mechanism	9	Mechanism used to implement the random allocation sequence (such as sequentially numbered containers), describing any steps taken to conceal the sequence until interventions were assigned	p 4
Implementation	10	Who generated the random allocation sequence, who enrolled participants, and who assigned participants to interventions	p 4
Blinding	11a	If done, who was blinded after assignment to interventions (for example, participants, care providers, those assessing outcomes) and how	p 4
	11b	If relevant, description of the similarity of interventions	NA
Statistical methods	12	Methods used to address each pilot trial objective whether qualitative or quantitative	p 7-8

**Results**			
Participant flow (a diagram is strongly recommended)	13a	For each group, the numbers of participants who were approached and/or assessed for eligibility, randomly assigned, received intended treatment, and were assessed for each objective	Figure 3 CONSORT flow diagram
	13b	For each group, losses and exclusions after randomisation, together with reasons	Figure 3
Recruitment	14a	Dates defining the periods of recruitment and follow-up	p 7
	14b	Why the pilot trial ended or was stopped	N/A
Baseline data	15	A table showing baseline demographic and clinical characteristics for each group	Table 1
Numbers analysed	16	For each objective, number of participants (denominator) included in each analysis. If relevant, these numbers should be by randomised group	Figure 3
Outcomes and estimation	17	For each objective, results including expressions of uncertainty (such as 95% confidence interval) for any estimates. If relevant, these results should be by randomised group	Table 3
Ancillary analyses	18	Results of any other analyses performed that could be used to inform the future definitive trial	p 10-12
Harms	19	All important harms or unintended effects in each group (for specific guidance see CONSORT for harms)	NA
	19a	If relevant, other important unintended consequences	NA
**Discussion**			
Limitations	20	Pilot trial limitations, addressing sources of potential bias and remaining uncertainty about feasibility	p 15
Generalisability	21	Generalisability (applicability) of pilot trial methods and findings to future definitive trial and other studies	p 15
Interpretation	22	Interpretation consistent with pilot trial objectives and findings, balancing potential benefits and harms, and considering other relevant evidence	p 14-15
	22a	Implications for progression from pilot to future definitive trial, including any proposed amendments	p 15
**Other information**			
Registration	23	Registration number for pilot trial and name of trial registry	p 16
Protocol	24	Where the pilot trial protocol can be accessed, if available	OSF data depository
Funding	25	Sources of funding and other support (such as supply of drugs), role of funders	p 16
Ethical approval	26	Ethical approval or approval by research review committee, confirmed with reference number	p 16

Reference: Eldridge SM, Chan CL, Campbell MJ, Bond CM, Hopewell S, Thabane L, et al. CONSORT 2010 statement: extension to randomised pilot and feasibility trials. BMJ. 2016;355. This is an Open Access article distributed in accordance with the terms of the Creative Commons Attribution (CC BY 3.0) license (http://creativecommons.org/licenses/by/3.0/), which permits others to distribute, remix, adapt and build upon this work, for commercial use, provided the original work is properly cited.

Table S2 *The template for intervention description and replication (TIDieR) checklist*

TIDieR Template for Intervention Description and Replication

Item	Item	Where located *	
		Primary paper	Other † (details)
	**BRIEF NAME**		
1.	Provide the name or a phrase that describes the intervention.	Title	
	**WHY**		
2.	Describe any rationale, theory, or goal of the elements essential to the intervention.	Page 2-3	
	**WHAT**		
3.	Materials: Describe any physical or informational materials used in the intervention, including those provided to participants or used in intervention delivery or in training of intervention providers. Provide information on where the materials can be accessed (e.g. online appendix, URL).		OSF data depository
4.	Procedures: Describe each of the procedures, activities, and/or processes used in the intervention, including any enabling or support activities.	Page 6-7	Figure 2
	**WHO PROVIDED**		
5.	For each category of intervention provider (e.g. psychologist, nursing assistant), describe their expertise, background and any specific training given.	Page 6	
	**HOW**		
6.	Describe the modes of delivery (e.g. face-to-face or by some other mechanism, such as internet or telephone) of the intervention and whether it was provided individually or in a group.	Page 4	
	**WHERE**		
7.	Describe the type(s) of location(s) where the intervention occurred, including any necessary infrastructure or relevant features.	Page 4-5	
	**WHEN and HOW MUCH**		
8.	Describe the number of times the intervention was delivered and over what period of time including the number of sessions, their schedule, and their duration, intensity or dose.	Page 6	Figure 2
	**TAILORING**		
9.	If the intervention was planned to be personalised, titrated or adapted, then describe what, why, when, and how.	Page 6	
	**MODIFICATIONS**		
10.‡	If the intervention was modified during the course of the study, describe the changes (what, why, when, and how).	N/A	
	**HOW WELL**		
11.	Planned: If intervention adherence or fidelity was assessed, describe how and by whom, and if any strategies were used to maintain or improve fidelity, describe them.	Page 10	Figure 4
12.‡	Actual: If intervention adherence or fidelity was assessed, describe the extent to which the intervention was delivered as planned.	Page 10	Figure 4

† If the information is not provided in the primary paper, give details of where this information is available. This may include locations such as a published protocol or other published papers (provide citation details) or a website (provide the URL).‡ If completing the TIDieR checklist for a protocol, these items are not relevant to the protocol and cannot be described until the study is complete.

Table S3 *Description of study outcome measures and their psychometric properties*

Outcome variables	Instruments	Description (Number of items, subscales)	Scoring	Psychometric properties	Cronbach's alpha (study data)	Post hoc power analysis
**Psychological need satisfaction**	The Psychological Need Satisfaction in Exercise (PNSE) scale (Wilson et al., 2006a)	- 18 items, 3 sub-scales<br>- 6-point Likert scales<br>- 6-items in each of the sub-scales: competence, autonomy, and relatedness	Sub-scale score = mean of the 6 items (range = 1- 6)<br>Total score = mean of the 18 items (range = 1- 6)	α >.90<br>(Wilson et al., 2006)	pre-test data: 0.871<br>post-test data: 0.898<br>combined: 0.929	0.68
**Exercise motivation**	Behavioral Regulation in Exercise Questionnaire (BREQ–3) (Markland & Tobin, 2004; Wilson et al., 2006b)	- 24 items, 6 subscales<br>- 5-point Likert scales<br>- 4 items in each of the sub-scales: amotivation, external regulation, introjected regulation, identified regulation, integrated regulation, intrinsic regulation	Sub-scale score = mean of the 4 items<br>Relative autonomy index: (amotivation× (−3)) + (external regulation× (−2)) + (introjected regulation× (−1)) + (identified regulation×1) + (integrated regulation×2) + (intrinsic regulation×3). Higher score = higher autonomous motivation<br>(range: -24 to 24)	Good factorial validity, reliability (Duncan et al., 2010)<br>Subscale α = 0.73–0.86<br>(Markland & Tobin, 2004)	pre-test data: 0.636<br>post-test data: 0.870<br>combined: 0.879	0.82
**Physical activity level**	Godin-Shephard leisure-time physical activity	- 3 items: the number of times in the past 7 days	Item weights: Strenuous x 9; Moderate x 5; Mild x 3	Percentage agreement between LTPA−Q &	combined: 0.634	0.40

	questionnaire (LTPA−Q) (Godin, 2011)	they have performed any strenuous, moderate, and mild physical activity of more than 15 minutes in duration	A total score ≥24 = Active<br>14 – 23 = moderately active<br><14 = sedentary	accelerometer classification coding: 70.8 %.<br>Sensitivity: 75.3%<br>Specificity: 58.5 %<br>(Amireault et al, 2015)		
**Quality of life**	Patient-Reported Outcomes Measurement Information System® – Preference (PROPr) (Dewitt et al., 2020)	- 31 items, 8 domains<br>- 5-point Likert scales<br>- 4 items each: physical function, anxiety, depressive symptoms, fatigue, sleep disturbance, ability to participate in social roles and activities, and pain interference<br>- 2 items: cognitive function<br>- 1 item: pain intensity (from 0 to 10)	Raw scores generated for each domain will be transformed into a T-score (mean = 50).<br>A utility PROPr score range can also be generated (range: −0.022 to 1.0)<br>Higher score = greater endorsement of the construct being assessed	Correlations between PROPr and the other quality of life summary measures ranged from 0.67 to 0.70 (Hanmer et al., 2018)<br><br>Convergent validity: r = 0.72 with EuroQol EQ-5D index value (EQ-5D) and Intraclass Correlation Coefficient (ICC) of 0.48 (Klapproth et al., 2022)<br><br>Construct validity supported in a previous study of patients in hemodialysis (Zhang et al., 2021).	pre-test data: 0.833<br>post-test data: 0.741<br>combined: 0.852	0.37
